# Supplementary figures and images for: Accessory Genomic Epidemiology of Cocirculating Acinetobacter baumannii Clones
Source: mSystems. 2021 Jul 20;6(4):e00626-21. doi: 10.1128/mSystems.00626-21 (PMC8407383; doi:10.1128/mSystems.00626-21)

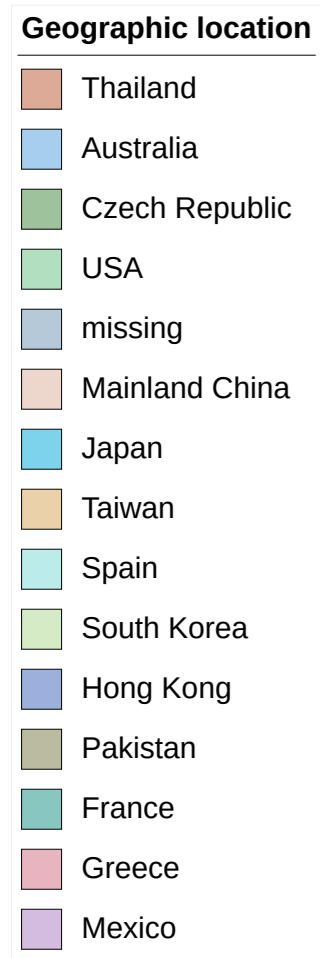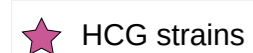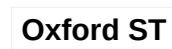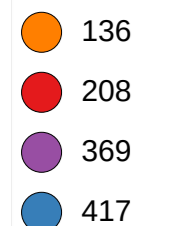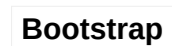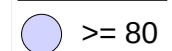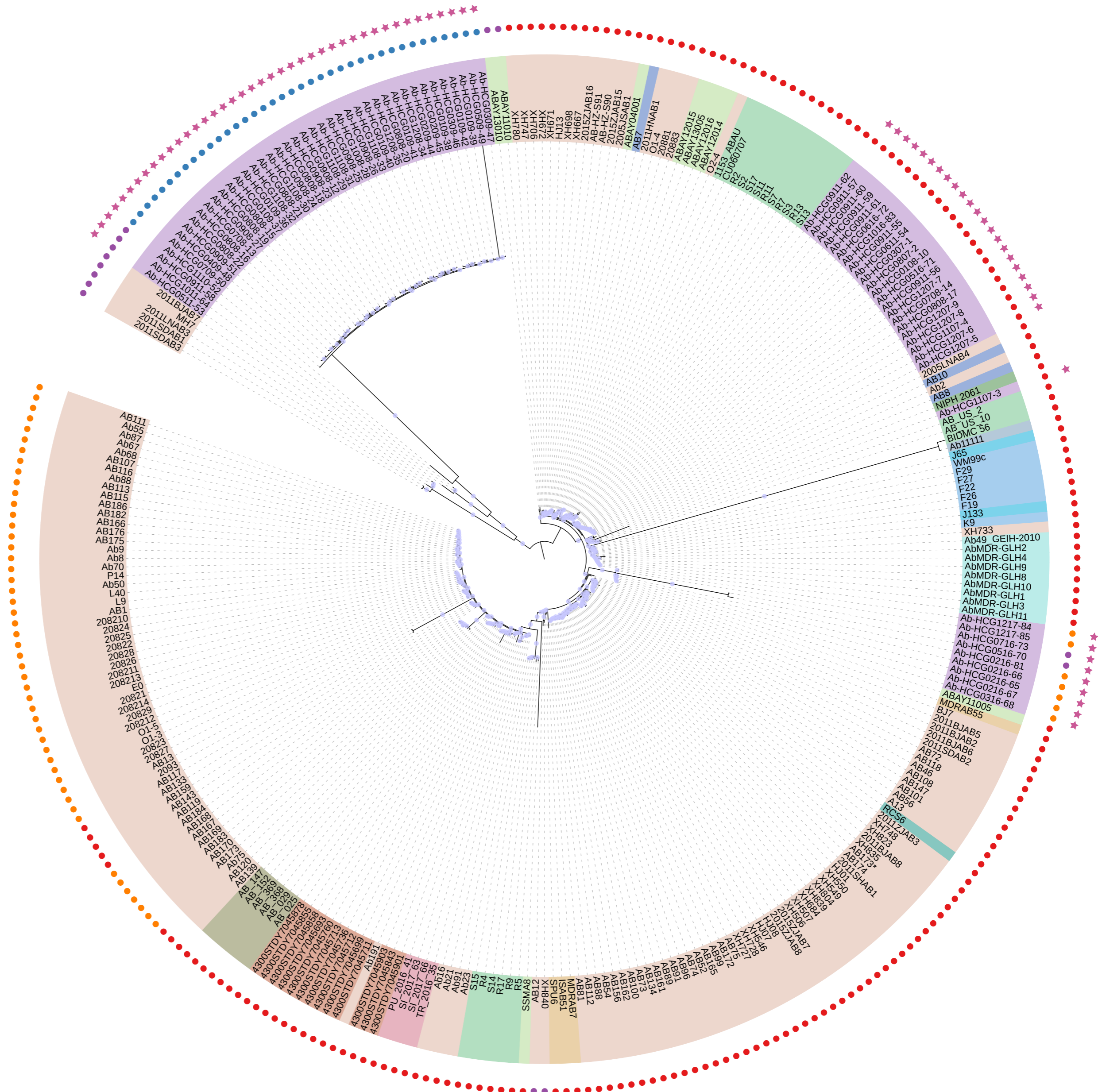

Supplement: FIG S2 [file msystems.00626-21-sf002.pdf]

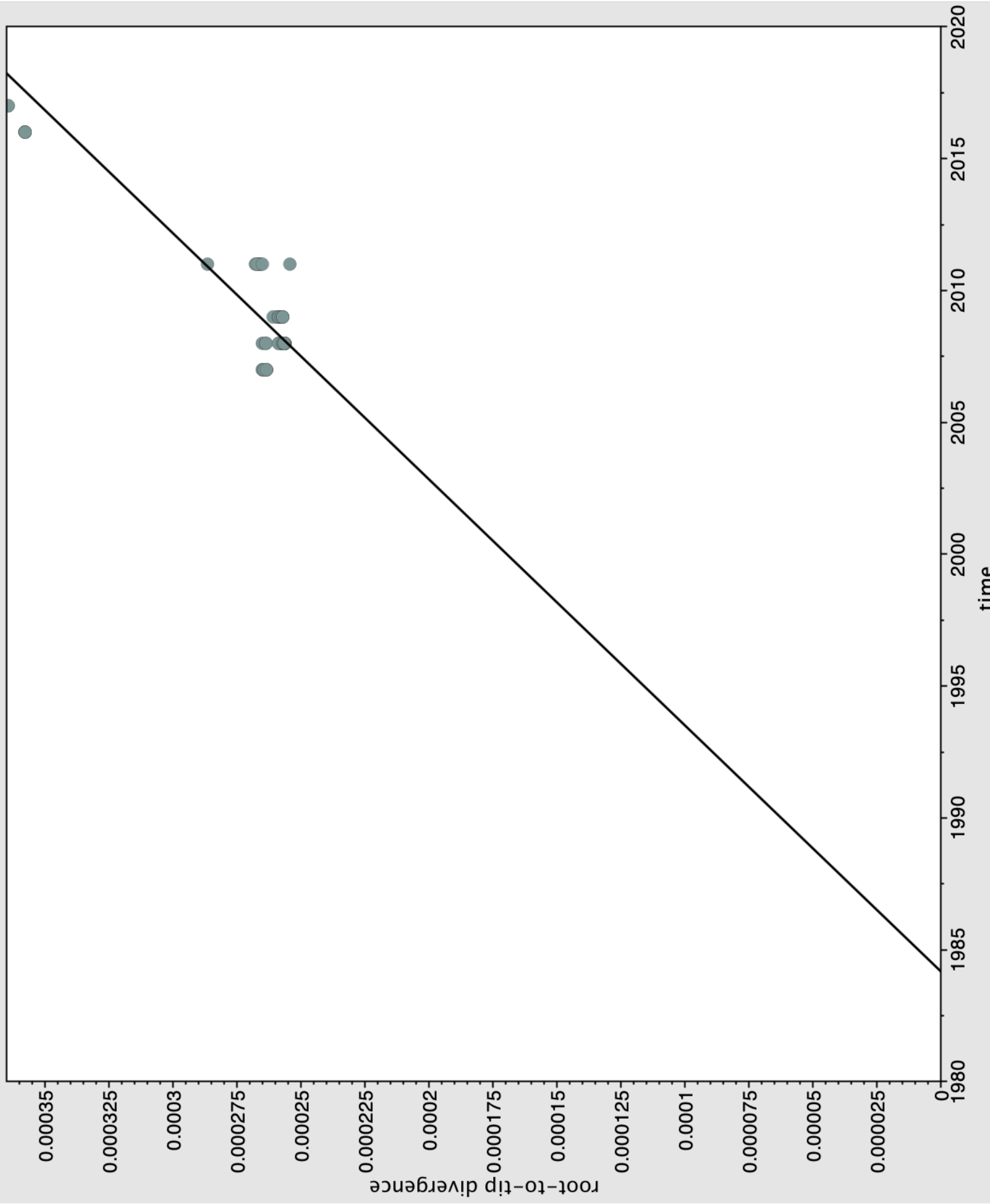

Supplement: FIG S1 [file msystems.00626-21-sf001.pdf]
